# Targeting the Large Subunit of Human Ribonucleotide Reductase for Cancer Chemotherapy

**DOI:** 10.3390/ph4101328

**Published:** 2011-10-13

**Authors:** Sanath R. Wijerathna, Md. Faiz Ahmad, Hai Xu, James W. Fairman, Andrew Zhang, Prem Singh Kaushal, Qun Wan, Jianying Kiser, Chris G. Dealwis

**Affiliations:** 1 Department of Pharmacology, School of Medicine, Case Western Reserve University, Cleveland, OH 44106, USA; 2 Center for Proteomics and Bioinformatics, Case Western Reserve University, Cleveland, OH 44106, USA

**Keywords:** ribonucleotide reductase, gemcitabine, clofarabine, cladrabine, P6, P7, Cyc 10, dATP, ATP, allosteric regulation, specificity cross-talk, nucleotides

## Abstract

Ribonucleotide reductase (RR) is a crucial enzyme in *de novo* DNA synthesis, where it catalyses the rate determining step of dNTP synthesis. RRs consist of a large subunit called RR1 (α), that contains two allosteric sites and one catalytic site, and a small subunit called RR2 (β), which houses a tyrosyl free radical essential for initiating catalysis. The active form of mammalian RR is an α_n_β_m_ hetero oligomer. RR inhibitors are cytotoxic to proliferating cancer cells. In this brief review we will discuss the three classes of RR, the catalytic mechanism of RR, the regulation of the dNTP pool, the substrate selection, the allosteric activation, inactivation by ATP and dATP, and the nucleoside drugs that target RR. We will also discuss possible strategies for developing a new class of drugs that disrupts the RR assembly.

## 1. Introduction

All cellular organisms require a balanced pool of deoxyribonucleoside triphosphates (dNTPs) to maintain their genomic integrity [1,2]. Ribonucleotide reductase (RR) catalyzes the rate limiting step of dNTP synthesis and provides the precursors essential for DNA replication and repair [3]. Depending on the organism, RR catalyzes the reduction of either ribonucleoside 5′-di- or triphosphates (NDP or NTP) to corresponding deoxyribonucleoside 5′-di- or triphosphates [4]. The reduction mechanism requires the generation of a thiyl radical (S•) in the active site of the enzyme. Three classes of RR have been described, based on their metal cofactors. These metal cofactors generate free radicals that lead to the formation of an essential S• radical in the active site of the enzyme [5,6]. Class I enzymes generate a tyrosyl free radical in a diiron-oxygen cluster located in the small subunit [7,8]. Class II enzymes directly generates the deoxyadenosyl free radical using Co-containing cobalamin as the cofactor [9]. Class III enzymes utilize 4Fe-4S type iron-sulfur clusters coupled to S-adenosylmethionine as their metal cofactor to generate a glycyl free radical [10]. In all three classes of RRs, these free radicals are ultimately delivered to a conserved active site cysteine residue to generate the S• radical [4].

### 1.1. Class I Enzymes

**Figure 1:**
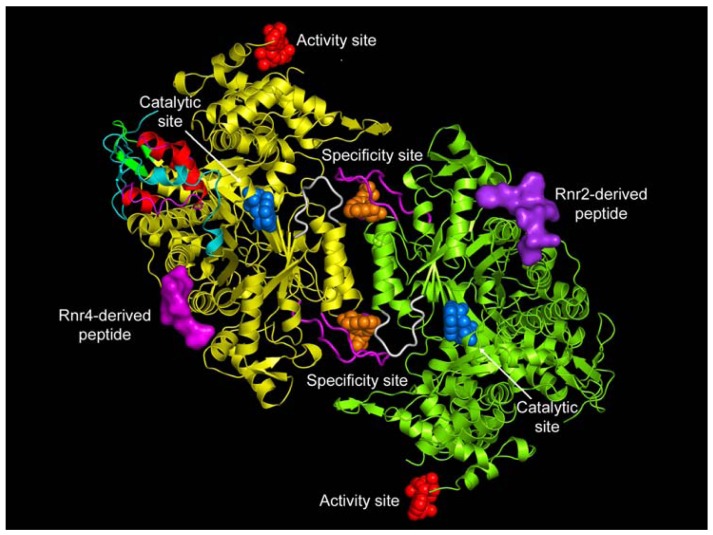
Class I Ribonucleotide reductase I. The specificity (S-site), catalytic (C-site), and activity (A-site) are shown as solid objects. Rnr2/Rnr4 peptides define the P-site. Loop 2 (white) and loop 1 (magenta) are close to the dimer interface. Reproduced with permission from *PNAS* [12]. Copyright (2006) National Academy of Sciences, USA.

Class I enzymes are expressed in almost all eukaryotic organisms, in some prokaryotes, and in certain viruses [4]. The functional form of eukaryotic Class I RRs have an active subunit composition of α_2_β_2_ and/or α_n_(β_2_)_m_ (where n = 4 or 6 and m = 1, 2 or 3). The reduction of the ribonucleoside diphosphate substrate occurs in the large α subunit, which also has two allosteric sites in addition to the catalytic site (C-site) (see Figure 1) [11,12]. The specificity site (S-site) determines which cognate substrate is reduced at the C-site, while binding of ATP or dATP to the activity site (A-site) determines the overall activity of the enzyme [13,14]. Class I enzymes are further divided into three subclasses based on the organization of the RR genes, subunit topology, and metal cluster assembly of the β subunit [1,15,16]. The small subunit (β) requires a metalo-cofactor for free-radical generation.

In Classes Ia and Ib, the generation of the tyrosyl free radical (Y•) in the β subunit is oxygen dependent. Therefore, these enzymes are expressed under aerobic conditions. The Class Ic enzymes do not generate Y•, but the synthesis of active metal cluster in the β subunit requires oxygen. Here we will be briefly describing the Class Ia enzymes. Readers are encouraged to consult references [1], [2] and [4] for additional details.

Class Ia enzymes are found in almost all eukaryotic organisms, in prokaryotes, and in viruses that infect eukaryotes [1]. The α subunit is encoded by the *nrdA* locus and the β subunit is encoded by the *nrdB* locus [17]. The β subunit possesses the diferric tyrosyl radical (Fe^III^Fe^III^-Y•) required for ribonucleoside diphosphate reduction [18]. The catalytic mechanism of Class Ia enzymes require the transport of this free radical over a 30-35 Å distance to the catalytic site [11,19]. At the end of each catalytic cycle, the active site cysteines become oxidized and their regeneration in Class Ia enzymes depends on the thioredoxin or glutaredoxin based system, which ultimately receives its reducing equivalents from NADPH [20]. The binding of either ATP or dATP to the ATP binding cone at the N terminus results in oligomerization of the subunit. The quaternary structures of class Ia enzymes are complex and have different subunit compositions with α_n_(β_2_)_m_ configuration, where n = 1, 2, 4 or 6: m = 1, 2 or 3 [21-26]. In *E. coli*, α and β subunits form α_4_β_4_ oligomers whereas in eukaryotes these subunits form both α_2_β_2_ and α_6_(β_2_)_m_ oligomers [21,26,27].

### 1.2. Class II Enzymes

Class II enzymes are found among prokaryotes and some lower order eukaryotes [4]. Class II RRs are composed of an α subunit encoded by the *nrdJ* gene [1]. The α subunit has only the C-site and the S-site [2]. The classes II RRs also lack the A-site and therefore are unable to be inhibited or activated by dATP or ATP, respectively. The free radical generation in Class II is oxygen independent and lacks a predefined subunit. The metalo-cofactor named 5-deoxyadenosylcobalamin binds to the α subunit and undergoes homolytic cleavage to yield the 5′-deoxyadenosyl radical [28,29]. The radical then propagates across a distance of 6 Å to the active site cysteine to generate the transient S• radical [30]. Two crystal structures of Class IIenzymes have already been solved [31,32].

### 1.3. Class III Enzymes

Class III enzymes are thought to be evolutionarily the most ancient RR enzymes [5]. They are found among some bacteriophages and in strict or facultative anaerobic bacteria [4]. The *nrdD* gene encodes the large α subunit and the *nrdG* gene encodes the small β subunit [33]. The structure of the α subunit of a T4 bacteriophage is the only structure currently available for this class and it shows the conservation of the 10-stranded β/α barrel containing the catalytic site [34]. The 4Fe-4S cluster reduces S-adenosylmethionine (SAM) in the β subunit to generate the glycyl radical on the α subunit [35]. Class III enzymes use a thiol independent reductant system during catalysis, hence it lacks the equivalent of Cys 439 (*E. coli* numbering). Instead, Class III enzymes depend on the oxidation of formate to CO_2_ in place of cysteine oxidation [36,37].

## 2. The Catalytic Mechanism

The sequence identity among the three classes of RR is less than 10% [2]. Their catalytic cores, however, show remarkable similarity, which is indicative of a common reaction mechanism for substrate reduction. The conserved catalytic domain among all three classes of RR contains the 10 stranded α/β barrel with the ‘RNR finger loop’ that harbors the thiyl free radical [11,12,31,32,38]. The key residues required for substrate reduction are structurally conserved between Class I and Class II enzymes. These residues include the two redox active site cysteines, the general acid/base catalyzing glutamic acid and its hydrogen bonding partner asparagine. During each catalytic cycle, the redox active cysteine pair undergoes oxidation, leading to the formation of a disulfide bond. Prior to the next turn-over, they are reduced. Class I and Class II enzymes achieve this through shuttling reducing equivalents from an external reductant system via two structurally conserved CXXC motifs present in their flexible C-terminal tails [39,40]. In turn the CXXC motif is reduced after each catalytic turn-over by the thioredoxin or glutaredoxin system [41-43]. In contrast, Class III RRs lack one of the corresponding cysteines in the redox pair in the catalytic site. Instead, Class III enzymes oxidize formate to carbon dioxide to generate reducing equvalents. The reaction mechanism of Class I enzymes are well studied in *E. coli* and will be presented briefly below [44,45].

**Figure 2:**
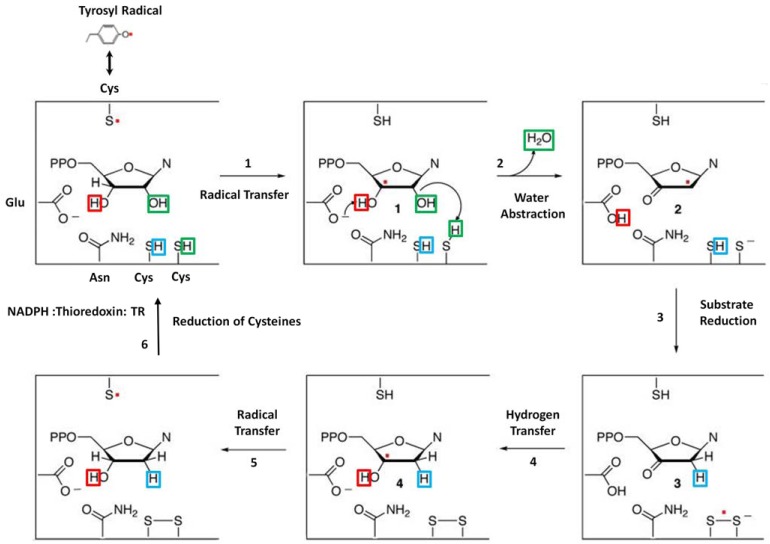
Catalytic Mechanism of Class Ia RR. See the text for a detailed description of the mechanism. Adopted from Zipse *et al.* [51] and modified. Copyright (2009) American Chemical Society.

Initiation of the catalytic cycle requires the formation of the holoenzyme complex and the binding of the cognate effector and substrate pair to the S-site and C-site. Once formed, the Y• is transferred from the β subunit to C439 of the α subunit some 35 Å, generating the thiyl radical [11,46,47]. This is followed by the abstraction of the 3′-hydrogen atom from the ribose sugar by the thiyl radical (C439), generating the 3′-carbon radical (Step 1) [48]. The formation of the 3′-nucleotide radical facilitates both the protonation of the 2′-hydroxyl group of the ribose ring by one of the catalytically active redox pair, (C225) and the deprotonation of the 3′-OH by the glutamate (E441). Subsequently, the 3′-nucleotide radical isomerizes to the 2′-nucleotide radical with the concomitant loss of a H_2_O molecule (Step 2). The second cysteine (C463) at the catalytic site then delivers the reducing equivalent to the 2′-nucleotide radical, leading to the generation of 3′-ketodeoxynucleotide and the disulfide radical anion (Step 3). When the free radical on the anion is then transferred back to the 3′-carbon of the deoxyribose sugar, the E441 now acts as a general base and protonates the 3′-ketodeoxynucleotide radical, yielding the 3′-hydroxynucleotide radical (Step 4) [45]. The free radical is then transferred back to the original free radical bearing cysteine (C439) thus regenerating the S• radical and the 2′-deoxyribonucleoside diphosphate (Step 5). In Class I RRs, the free radical is transferred back to the β subunit. During this process, the redox cysteine pair undergoes oxidation and regenerates before the next catalytic cycle [49]. The latter is achieved by the reducing equivalents carried by the RR1 C-terminus and the NADPH-thioredoxin/glutaredoxin based reductase system (Step 6) (Figure 2) [39,50].

## 3. Regulation Of RR

RR functions as a principal regulator of cellular dNTP pools. The maintenance of a balanced dNTP pool is a prerequisite for high fidelity DNA replication and repair following DNA damage [1]. Therefore, RRs are subjected to multiple modes of regulation: (1) allosteric regulation [13], (2) subunit oligomerization [22], (3) transcriptional regulation of RR genes [52], (4) binding of small protein inhibitors [53,54], (5) subunit compartmentalization [55,56], (6) cofactor assembly and free radical generation [6]. Some of these mechanisms are conserved across species and other regulatory mechanisms are organism specific. Only the major and commonly conserved mechanisms in mammalians will be discussed in this review.

### 3.1. Allosteric Regulation

#### 3.1.1. Substrate Selection

A remarkable feature of the RRs is their ability to reduce four different NDP or NTP using an intricate mechanism of substrate selection [13,57]. The RRs accomplish this by an elegant allosteric mechanism requiring the coordination of two allosteric effector binding sites and the catalytic site (Figure 1). The basic mechanism of substrate selection was first elucidated for prokaryotes [13]. Based on these studies, it was proposed that the large α subunit contains two separate allosteric effector binding sites, one regulating the overall activity (A-site) and the other regulating the substrate specificity (S-site). The A-site binds both the allosteric activator ATP and the allosteric inhibitor dATP. The S-site binds dGTP, TTP, ATP and dATP. ATP or dATP bind at the S-site and selects either CDP or UDP to be reduced at the C-site. The product dUDP is subsequently dephosphorylated to dUMP and further metabolized by thymidylate synthetase to form TMP, which is then phosphorylated to TTP [58]. TTP binds at the S-site and selects for GDP to be catalyzed at the C-site. Nucleotide diphospshate kinase (NDK) converts the dGDP to dGTP, which in turn binds the S-site and selects for ADP substrate reduction. The conversion of dADP to dATP is also accomplished by NDKs.

The relatively high affinity of dATP compared to ATP [22,59] enables it to compete and displace ATP from the A-site leading to the inhibition of the enzyme activity. Thus, the coordination of effector and substrate binding sites and the relative affinities of ATP and dATP maintain the balanced dNTP pool in the cell. Some of these features are shared by both class I and class II enzymes. In contrast, Class III enzymes use a slightly different set of substrate selection rules. The A-site is known as the pyrimidine site and can bind either ATP or dATP. Binding of ATP to the pyrimidine site stimulates reduction of pyrimidine ribonucleotides. The purine site resembles the S-site in Class I and Class II enzymes. dGTP and TTP bind at the purine site and selects for ATP or GTP at the C-site, respectively. However, the binding of dATP to either the purine or pyrimidine site is always inhibitory.

The molecular basis for substrate selection are described in two studies based on the class II and class I structures [12,32]. Prior to the studies, two important loops called loop 1 (residues 245 to 260) and loop 2 (residues 285 to 295) (Figure 1) were identified using the *E. coli* X-ray structure [49]. Nucleoside or deoxynucleoside triphosphate effector binding at the S-site is a prerequisite for the dimerization of the large subunit, which is essential for substrate selection. This is because allosteric communication occurs between subunits involving loop 2 (Figure 1), which connects the S-site on one subunit with the C-site of the adjacent subunit.

**Figure 3:**
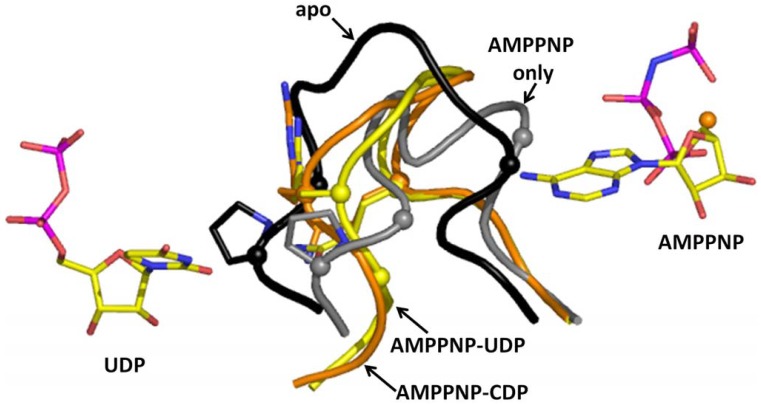
Loop 2 movements upon binding of effectors: Substrate (CDP) and loop 2 and effector (AMPPNP) are shown for AMPPNP-UDP. Loop 2 is shown for apo (black), AMPPNP only (gray), and AMPPNP-CDP(orange). Reproduced with permission from PNAS [13]. Copyright (2006) National Academy of Sciences, USA.

Now we will describe the findings from the yeast RR1 structure. In the apo enzyme, loop 2 occupies a position that sterically restricts substrate binding. This observation is consistent with biochemical data which show that only 10% of the activity is retained without effector binding [22,40]. When effectors bind at the S-site, loop 2 moves away from the C-site towards the S-site, thereby creating space for substrates to bind (Figure 3). Once the substrate binds the C-site, loop 2 shifts partially back towards the C-site. We called the elegant communication between the S-site and the C-site “specificity crosstalk”.

**Figure 4:**
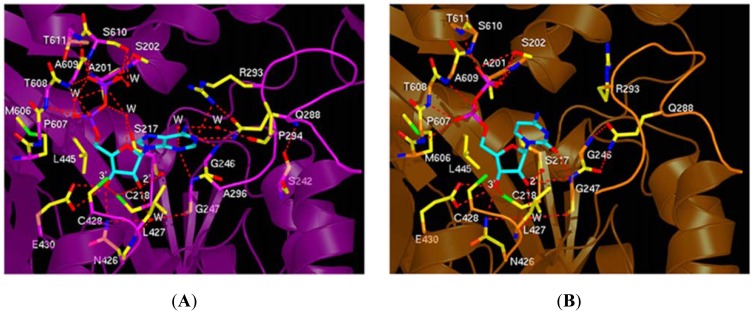
Substrate selection. (**A**) ADP binding at the C-site and (**B**) CDP binding at the C-site. The key residues on loop 2 required for substrate selection are Q288 and R293 are to the right. The catalytic residues C218, C428, N426 and E428 are also shown binding to the ribose moiety. Reproduced with permission from *PNAS* [13]. Copyright (2006) National Academy of Sciences, USA.

The substrate selection rules that were first proposed by Brown and Reichard [13,60] at the molecular level are maintained by specific interactions made between residues of loop 2 and the substrate. In particular, Arg 293 and Gln 288 are crucial for substrate recognition. Specifically, these residues appear to be crucial for ADP selection (Figure 4A). Arginine 293 forms a hydrogen bond and makes stacking interactions with the adenine ring, while Gln 288 forms a hydrogen bond. The importance of Arg 293 and Gln 288 in the yeast enzyme was recently shown in a mutagenesis study to be synthetically lethal [61]. In case of GDP, CDP, and UDP selection, these residues only make van der Waal's contacts (Figure 4B). Several water molecules also were shown to be important for substrate selection.

#### 3.1.2. ATP/dATP Induced Subunit Oligomerization

**Figure 5:**
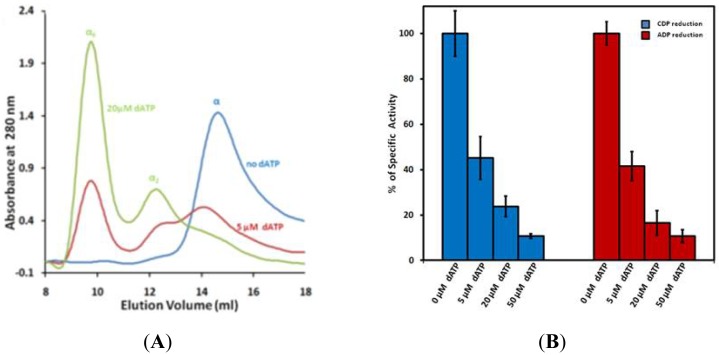

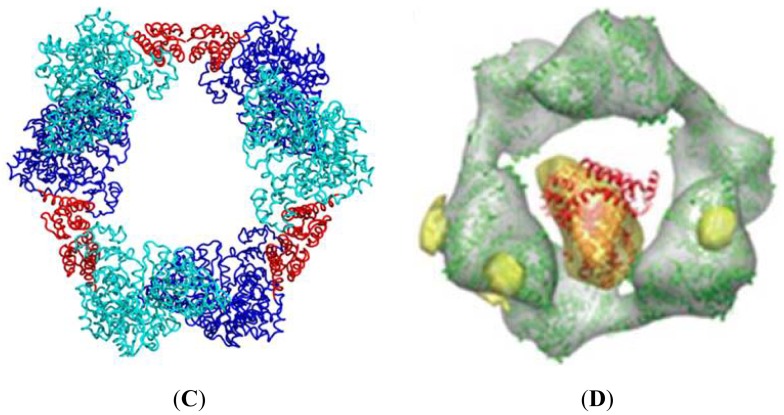
Characterization of dATP hexamer: (**A**) human RR1(hRR1) was tested for its ability to form hexamers in the presence of varying concentrations of dATP. No oligomers were observed in the absence of dATP (blue trace) and a mixed population of monomers, dimers, and hexamers at a dATP concentration of 5 μM (red trace). At 20 μM dATP, the hexamers are the dominant species, with a small amount of dimer (green trace). (**B**) The specific activity of the wild type enzyme decreased with increasing concentration of dATP. Activities for [1H] CDP reduction (blue) and [14C] ADP (red) reduction is shown. (**C**) Hexameric packing of RR1 based on the low-resolution X-ray crystal structure of the ScRR1 hexamer. ScRR1 monomers are colored in forest green and limon or blue and cyan. All the four-helix ATP-binding cones are colored in red. (**D**) Model of the α_6_●ββ′●dATP holo complex Reproduced with the permission from NSMB 2011 [26].

Both dATP and ATP regulate RR by altering its oligomeric state in a concentration-dependent manner. Initial studies with the mouse RR (mRR1) showed that dATP forms inactive tetramers while ATP forms active hexamers [22]. Later studies with Gas-phase Electrophoretic-Mobility Macromolecule Analysis (GEMMA) revealed that RR1 forms hexamers with either dATP or ATP, but not tetramers [21]. GEMMA experiments also indicated that these hexamers can associate with the RR2 subunit to form α_6_β_2_ complexes which are either active or inactive, depending on ATP or dATP binding [21]. In another study, the widely used cancer inhibitor gemcitabine was shown to induce the formation of a stable α_6_β_6_ RR complex [25]. Recently, we demonstrated that hexamerization is a prerequisite for the inhibition of RR1 (Figures 5A,B) and dimers of RR1 are not inhibited by dATP. The structure of the dATP-hexamer allowed visualization of subunit packing for the first time (Figure 5C). The cryo-EM structure showed that the dATP holoenzyme has a subunit composition of α_6_ β_2_ (Figure 5D). In the same study, based on site-directed mutagenesis we showed that the ATP hexamer adopts a different interface than dATP hexamer. Hence, there must be structural differences between the ATP hexamer and the dATP hexamer for one to be active while the other to be inactive.

### 3.2. Transcriptional Regulation of RR

The activity of RR is cell cycle dependent. RR1 protein level becomes highest during the S-phase of the cell cycle because of transcriptional induction but remains low during G1 and G2-phase [62]. In contrast, R2 protein levels are undetectable in G0 and G1-phase but rise dramatically in the S-phase after transcriptional induction [62]. RR activity reaches its maximum level during the S-phase of the cell cycle, thus providing the dNTP required for DNA replication. Mammalian cells also contain an additional RR subunit known as p53R2, which is induced after DNA damage [63,64]. p53R2 subunit is expressed in quiescent and post mitotic cells where it combines with RR1 to synthesize dNTPs required for nuclear DNA repair and mitochondrial DNA replication [65].

## 4. RR1 the Drug Target

RR is an attractive target for both cancer chemotherapy and antiviral therapy [66]. During the last few decades, a considerable amount of effort has been devoted to developing specific and novel inhibitors of this enzyme [67]. This review will focus on both inhibitors currently in use and others in development for cancer chemotherapy. We will discuss these inhibitors under three broad categories: translational inhibitors, inhibitors of the large subunit of RR, and inhibitors of the small subunit of RR.

### 4.1. Translational Inhibitors

Translational inhibitors of RRs are complimentary oligonucleotides that bind to the mRNA of either RR1 or RR2. Once complexed, these oligonucleotides either block translation or degrade the mRNA by activating RNase H. GTI-2040 and GTI-2501 are two such promising 20-mer phosphorothioate oligonucleotides that have undergone clinical trials [68]. GTI-2501 targets the coding region of RR1 and reduces both mRNA levels and RR1 protein levels in a dose-dependent manner. Both *in vitro* and *in vivo* studies have shown that GTI-2501 significantly inhibits the growth of various human cancer types [69].

### 4.2. Inhibitors of the Large Subunit of RR

Four druggable sites have been identified to date for ribonucleotide reductase I (Figure 1). They are: (1) the A-site, (2) the S-site, (3) the C-site and (4) the P-site. The first three sites are nucleoside and deoxynucleoside binding sites, while the fourth is the peptide binding site. The A-site and S-site bind ribonucleoside/deoxyribonucleoside triphosphates while the C-site binds ribonucleoside diphosphates. Most of the nucleotide-based drugs that target ribonucleotide reductase obtain their potency by binding at either the A-site or the C-site. Most of the analogs that bind the A-site often bind the S-site. Now we will briefly describe the drugs and their modes of mechanism when binding to the A-site, C-site and P-site of ribonucleotide reductase.

#### 4.2.1. A-Site Analogs

Fludarabine [70], cladribine [71,72], and clofarabine [73] are clinically used drugs and their metabolites target the A-site of RR1. These appear to be non-covalent inhibitors that bind the A-site. Of these three, the best characterized is clofarabine, which is used to treat childhood leukemias [73-75]. It appears that the clofarabine triphosphate is a good analog of dATP. Like dATP, which hexamerizes RR1, clofarabine also has been shown to be able to hexamerize RR1 [76]. In this study, Stubbe and coworkers showed that the clofarabine is not an irreversible inhibitor. They also showed that clofarabine triphosphate inhibits hRR1 with a K_i_ equal to 40 nM towards the A-site. After initial inactivation, however, the enzyme recovers 50% of its activity. Furthermore, in the study clofarabine diphosphate was shown to have a slightly lower K_i_ of 17 nM in the C-site and also induced RR1 hexamers. As previously mentioned regarding allosteric regulation by ATP and dATP, hexmerization is important for both activation and inactivation of the enzyme. In particular, dATP at physiological concentrations causes RR1 to hexamerize. Using site directed mutagenesis, we have shown that dimers of hRR1 cannot be inhibited by dATP [26]. Hence, nucleoside analogs that retain the ability to hexamerize the RR1 subunit similarly to that of dATP are likely to be potent inhibitors of ribonucleotide reductase. Cladribine and fludarabine have not been subject to such intense studies as clofarabine. Therefore, it is difficult to say precisely if they too will behave like clofarabine.

#### 4.2.2. C-Site Analogs

The most well studied analog that binds the C-site is gemcitabine [25,77]. Gemcitabine is a billion dollar drug that is a major component of standard chemotherapies for treating various cancers such as lung and pancreatic carcinomas [78,79]. Gemcitabine, an analogue of deoxycytidine (2′-2′-difluorodeoxycytidine, F_2_dC), is sequentially phosphorylated to the 5′-monophosphate (F_2_dCMP) by deoxycytidine kinase, and to difluorodeoxycytidine 5′-diphosphate (F_2_dCDP) by uridylate-cytidylate monophosphate kinase (UMP/CMP kinase) [80]. However, phosphorylation of F_2_dCMP by UMP/CMP kinase has been controversial, since the metabolites levels of gemcitabine remain unaffected in cell lines overexpressing or underexpressing this enzyme [81]. In the presence of reductants, F_2_dCDP covalently modifies RR1. In the absence of reductants, with pre-reduced RR1 and RR2, inhibition occurs from the loss of the tyrosyl radical in RR2 [25]. F_2_dCDP inactivates human RR by generating a tight α_6_β_6_ complex. F_2_dCDP has recently been shown to inhibit p53R2 (β′), but unlike in α_6_β_6_, the α_6_β′_6_ complex appears to be much weaker one [77]. Inhibition of RR by F_2_dCDP leads to reduction of the pool of dNTPs available for DNA synthesis, presumably favoring incorporation of the gemcitabine triphosphate metabolite by DNA polymerase α into growing DNA strands. [82].

Radiation sensitization by gemcitabine has been shown to correlate with dATP depletion through RR inhibition and S-phase accumulation [83]. Schewach and colleagues hypothesized that radio-sensitization to F_2_dCDP is due to nucleotide misincorporations in the presence of dNTP pool imbalances augmenting cell death following irradiation. The misincorporation rates become significant when mismatch-repair deficient cells were irradiated and treated with F_2_dCDP. The inhibition of ribonucleotide reductase is thought to be responsible for the dNTP misincorporation. The disruption of allosteric regulation of RR can lead to dNTP pool imbalances. In the case of F_2_dCDP, nucleotide pool imbalances probably occur through the inactivation of RR at the catalytic site and disrupting the allosteric communication between the specificity and catalytic sites. The latter is described below.

Although, there are no structural data for the quaternary structure of RR in complex with gemcitabine, we were able to determine the initial interactions of F_2_dCDP at the C-site using the yeast RR1. Now we will summarize the findings of this study [84].

**Figure 6:**
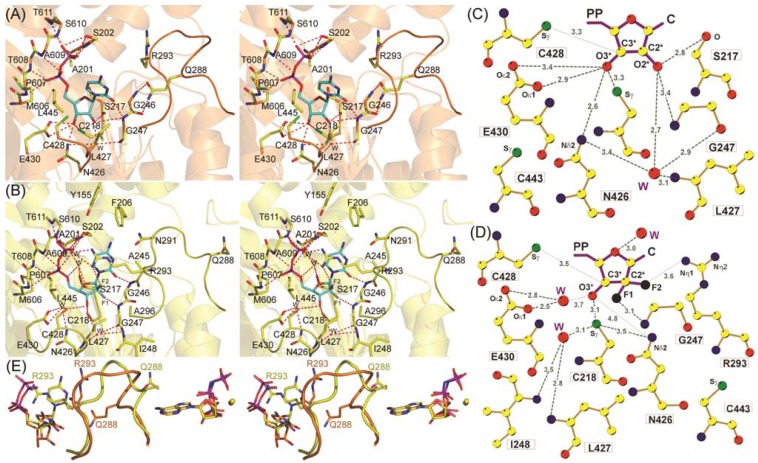
Catalytic site interactions of CDP and F_2_dCDP taken from Xu *et al.*, PNAS 2006. (**A**) Stereo view of CDP (orange). Interacting atoms: oxygen, red; nitrogen, blue; phosphate, magenta; sulfur green; substrate carbons, cyan; protein non-Cα, yellow; Cα, as secondary structure, orange. (**B**) Stereo view of F_2_dCDP. Interacting atoms are colored as in (*A*) above except sulfur orange; Cα carbons, as secondary structure, yellow; fluorines, grey. (**C**) Ligand plot of CDP ribose interactions. Colors are as in Figure 6(A), except that carbons are yellow and fluorine, black. (**D**) Ligand plot of F_2_dCDP interactions. The van der Waals contact to L427 is omitted for clarity. (**E**) Stereo view of loop 2 superposition of AMPPNP-CDP (orange) and AMPPNP-F_2_dCDP (yellow). Substrate/inhibitor is seen on the left and the effector is on the right. Reproduced with permission from *PNAS*. Copyright (2006) National Academy of Sciences, USA [84].

It is interesting to note that while F_2_dCDP differs from CDP only by substitution of two fluorines for the hydroxyl group and the hydrogen atom bonded to the 2′C of the ribose ring, F_2_dCDP adopts a different conformation when binding to RR1. We showed that in the AMPPNP-CDP structure the 2′ and 3′ OH of the ribose are close to the catalytic N426 and E430, C428 where the thiyl radical is generated on RR1 by a series of coupled electron and proton transfers [40], and C218 of the reduced catalytic redox pair (C218 and C443). In contrast, in the AMPPNP-F_2_dCDP structure, we observed that the ribose and especially the base of F_2_dCDP appear to bind higher in the pocket (Figures 6A,C,E), such that the 2′ carbon and the two fluorines of the F_2_dCDP ribose bind near the location of C2, N3, and O2 of CDP's cytidine base in the AMPPNP-CDP structure (Figure 6E). We observed that the F_2_dCDP ribose is displaced by an average of 2.3 Å, and its cytidine base by an average of 3.8 Å compared to those of CDP. F_2_dCDP's unique mode of binding places its ribose further away from the active site residues N426 and E430 (Figures 6A-D).

We showed that the differences observed between CDP and F_2_dCDP binding was due to the unique chemical properties of the geminal fluorine atoms, which are more hydrophobic and yet retain the ability to form hydrogen bonds [85]. It appears according to this study that geminal fluorines can form hydrogen bonds to donor nitrogen atoms with hydrogen bond lengths ranging from 3.0–3.6 Å and C-F-N angles ranging from 60°–180°. In the AMPPNP-F_2_dCDP structure we note that the F2 fluorine that replaces the hydrogen atom forms a weak hydrogen bond (3.6Å) with the guanidinium group of R293 from loop 2 with a C-F-N angle of 142°. Furthermore, this F2 fluorine has been shown to hydrogen bond an arginine in deoxycytidine kinase, which phosphorylates gemcitabine [86]. The F1 geminal fluorine forms a hydrogen bond (3.1Å) with the amide nitrogen of G247 with a C-F-N angle of 141°. However, in the AMPPNP-CDP complex CDP does not interact with R293 while the 2′ OH forms a longer (3.5 Å) hydrogen bond to the amide nitrogen of G247. We observed that the hydrogen bond between the 2′ OH of CDP and the CO of S217 is missing in the AMPPNP-F_2_dCDP structure. Also, F_2_dCDP's F1 makes a close van der Waals contact (3.3 Å) with CD2 of L427, possibly due to the more hydrophobic nature of fluorine; the corresponding distance in the AMPPNP-CDP complex is 4.2 Å. As for the catalytic residues, we observe the 3′ carbon of F_2_dCDP is within 3.5 Å of C428, and the 3′ OH of GemdP is 3.1 Å from the C218. Moreover, it is interesting to note that like in the AMPPNP-CDP structure the OH of Y741 that is in the free radical relay pathway is within 3.5Å of the Sγ of C428 in the AMPPNP-F_2_dCDP structure. It is important to note that these distances should still permit mechanism based inhibition, which requires abstraction of the F_2_dCDP's 3′ hydrogen atom by a thiyl radical generated at C428 by a series of coupled proton and electron transfers from Y183• of RR2 [44]. Our study illustrates the unique interactions that fluorine atoms can make, where chemical space truly invades biological space.

As previously mentioned, the clofarabine diphosphate metabolite is a potent inhibitor of ribonucleotide reductase [76]. It too binds at the C-site and the mode of inactivation is via reversible inhibition inducing hexmerization of the large subunit of ribonucleotide reductase. There are other examples of C-site inhibitors. In a rational drug design effort, we were able to modify the 2′ hydroxyl of the ribose ring with a hydroxyethylene moiety [87]. The rationale behind the design involved the observation of a water molecule bound at the active site of the yeast enzyme [84]. The hydroxylethyl moiety was to mimic the water molecule and displace it upon binding. The crystal structure of the 2′ hydroxylethylene adenine diphosphate was shown to displace the water molecule and bind in its place. This study shows the potential for designing new C-site inhibitors.

In a theoretical study by Pereira and *et al.*, report a possible mechanism for RR inhibition by F_2_dCDP in the absence of reductants [88]. This mechanism is very similar to the natural substrate reduction pathway and only deviates from the natural course after the formation of the well-known disulphide bridge. They propose that the deviation is caused by the F atom present in this inhibitor. Based on this mechanism the essential radical in RR2 is lost, along with the enzyme catalytic activity. A more comprehensive review of the theoretical work conducted on ribonucleotide reductase is given elsewhere [89].

#### 4.2.3. Targeting the Peptide Binding Site (P-SITE)

In 1990, Cooperman and co-workers demonstrated that mRR can be inhibited by competitive binding at the mRR1 subunit by the P7 heptapeptide (N-AcFTLDADF), which corresponds to the C-terminus of the RR2 subunit [90]. It was shown by transfer-NOE NMR studies that P7 bound to mRR1, adopting a reverse-turn structure for residues 2–5, TLDA [91,92]. Furthermore, these results, and related structure-function [93,94] and modeling [95] studies, based on the then known structure of *E. coli* RR2 (EcRR2) C-terminal peptide (EcRR2pep) bound to *E. coli* RR1 (EcRR1) [11], led to the notion that P7 C-terminal peptide binding occurs at two contiguous subsites in mRR1, denoted site 1 (for the N-terminal Phe residue) and site 2 (for the C-terminal Phe residue) [95]. It was thought that the site 1 subsite, accommodating the N-terminal portion of the peptide, was posited to be broad, shallow, hydrophobic, and not strongly sequence specific, while the F7 subsite 2, which accommodates the C-terminal portion, was posited to be narrow and deeper, with very high specificity for the ultimate C-terminal residue. Furthermore, in previous studies, specific locations for the site 1 and site 2 subsites within mRR1 were proposed based on homology with the EcRR1:EcRR2pep complex structure [11].

The Cooperman group targeted site 1 and site 2 to conduct a series of directed minilibrary screening studies having the goal of developing peptide-based inhibitors of mRR with high affinity for mRR1 [96]. Based on this work one important result was the identification of the peptidomimetic, ^1^Fmoc(Me) PhgLDChaDF^7^, denoted P6, which has a K_i_ for mRR1 dimer of 310 nM, some 8-fold lower than the corresponding value for P7.

**Figure 7:**
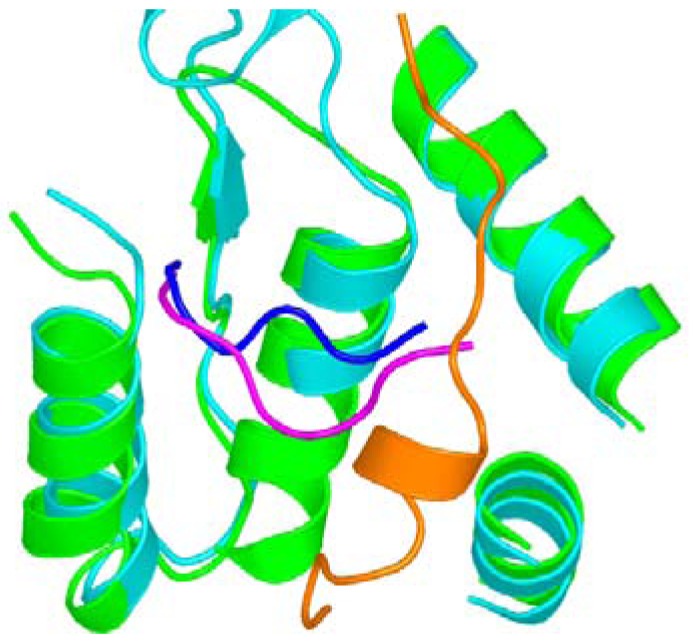
View of the structural comparison of ScRR2, ScRR4 and EcRR2 peptide binding. Carbon atoms for ScRR2 peptide (magenta), ScRR4 peptide (blue) and EcRR2 peptide (orange). Nearby helices are drawn from the ScRR1-ScRR2 complex (green) and EcRR1-EcRR2 complex (cyan). The figure was reproduced with permission from *PNAS* [12]. Copyright (2006) National Academy of Sciences, USA.

In another study, we reported the first structure of a eukaryotic RR1, *S. cerevisiae* R1 (ScRR1) [12,84], in which the ScRR2 C-terminal peptide (ScRR2pep) bound to ScRR1 at a region consisting of residues that are highly conserved between yeast, mouse, and human RR1s (but not among prokaryotes), suggesting that the mode of RR1-RR2 binding is conserved among eukaryotes [95]. We used a nonapeptide derived from the ScRR2 C-terminus for making the ScRR1-ScRR2pep complex. In this study only the last seven amino acid residues could be located in the structure. In the same study, we also solved the structure of ScRR1 in complex with the C-terminal peptide derived from ScRR4 (ScRR4pep). Here only the last six amino acid residues were observed [84]. To our surprise, we observed that the mode of ScRR2pep binding to ScRR1 was markedly different from that previously reported for the EcRR2pep-EcRR1 complex [84]. We noted that when the ScRR1 and EcRR1 structures are superposed, ScRR2pep binds essentially at a right angle with respect to EcRR2pep, and in a non-helical conformation (Figure 7).

##### 4.2.3.1. P6 and P7 Mammalian RR Inhibitors

Both P6 (^1^Fmoc(Me)PhgLDChaDF^7^) and P7 (Ac-^1^FTLDADF^7^) are potent inhibitors of mRR and ScRR with an IC_50_ in the low μM range. Note that P7 inhibits mRR somewhat more strongly than ScRR (8.9 μM *vs.* 31 μM), in accord with an earlier report by Xu *et al.* using a crude ScRR preparation [93]. P6 is nearly equipotent toward mRR or ScRR (1.9 μM *vs.* 2.6 μM). In that study we noted that the general similarity in inhibition values toward both enzymes may suggest that the binding of peptide and peptidomimetic inhibitors to ScRR1 shown in this work provide a good model for how such inhibitors bind to mRR1.

##### 4.2.3.2. P7's Mode of Binding

**Figure 8:**
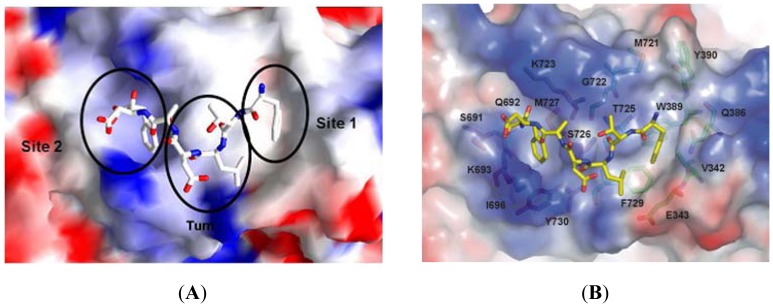
Binding of P7 to ScRR1. (**A**) P7 binds at subsites 1 and 2 connected by a reverse turn (**B**) P7-yellow where subsite 1 is to the right involving F1 and subsite 2 is to the left involving D6F7. ScRR1 binding site is depicted as a surface. The figure was reproduced with permission from Xu *et al.* [97]. Copyright (2008) American Chemical Society.

We observed that the peptide adopts a non-standard reverse turn involving residues 2–5 when binding to ScRR1. Also, P7 binds ScRR1 at the periphery at two surface subsites 1 and 2 (Figure 8A), orthogonal to and separated by the helix αI. We showed that subsite 1 is positioned near α13 and α D, and subsite 2 is positioned near α H (Figure 8B) [84].

We showed that subsite 1, consisting of V342, E343, Q386, W389, L393, M721, G722, T725, and F729, is broad and extremely hydrophobic (Figure 8B), and anchors the side chains of the N-terminal F^1^ and L^3^ residues. F^1^ stacks strongly with W389 and also interacts with V342 of α 13 and T725 of α I while L^3^ packs edge-to-face with F729 of αI. Moreover, the highly positively charged surface of subsite 2 contains residues S691, Q692, K693, I696, K723, S726, M727, and Y730 (Figure 8B). We observed that the side chain groups of D^6^ and F^7^ bind in subsite 2 with the negative charge of the carboxylate terminal forming two hydrogen bonds with S691 and Q692, and one long range ion pair interaction with K723. Moreover, another ion pair interaction is formed between D^6^ and K693 (Figure 8B). Note that the interior of subsite 2 is narrow and quite hydrophobic, accommodating the side chain of F^7^.

##### 4.2.3.3. P6's Mode of Binding

**Figure 9:**
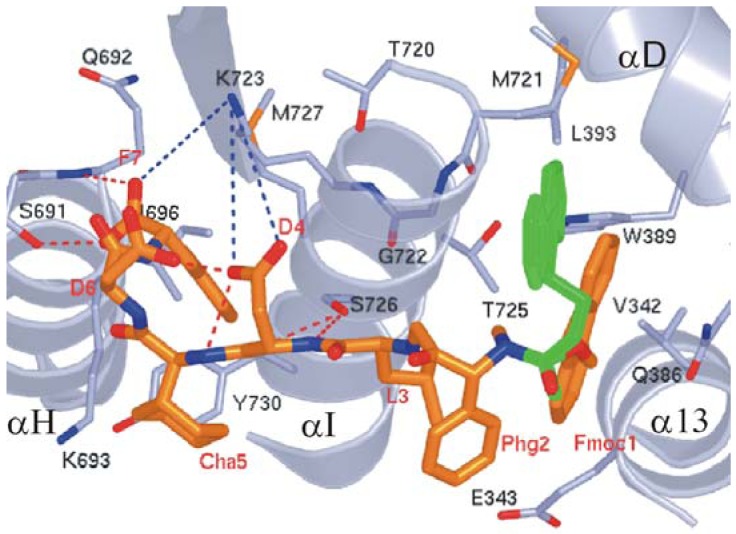
P6 binding to ScRR1. P6-orange, The figure was reproduced with permission from Xu *et al.*, [97]. Copyright (2008) American Chemical Society.

Based on the study by Xu *et al.* [97], our X-ray structure clearly shows that the P6 peptide binds ScRR1 with a partially extended conformation (Figure 9), lacking the reverse turn found in ScRR1-bound P7 (Figure 8). We observed that there are also main-chain conformational differences between position 4 and 6. In this study we attributed the altered P6 binding mode to the substitution of non-standard residues ^1^Fmoc (Me), Phg, and Cha at positions 1, 2 and 5, respectively. We showed that the ScRR1-bound structures of P7 and P6 superpose with an RMSD of 1.88 Å, further demonstrating their main-chain conformational differences (Figures 8B and 9).

Previously, the Cooperman group had proposed that the addition of the Fmoc group at the N-terminus would improve binding due to the contributions of the hydrophobic interactions made with mRR1 at subsite 1 [95,98]. Although the resolution of the P6 structure (2.5 Å) in this study is insufficient to conduct occupancy refinement, we saw electron density for two conformations based on comparisons of B-factors. The Fmoc conformation binding at the hydrophobic subsite 1 is likely to have the greater occupancy, while the minor conformation partially points to solvent. The major conformation of Fmoc makes several intramolecular and intermolecular hydrophobic interactions (Figure 9). We observed that one of the six-membered rings of Fmoc binds ScRR1 almost identically to the F^1^ side chain of the P7 inhibitor, while the second six-membered ring makes additional hydrophobic interactions with the indole ring of W389 and the side-chain of L393. We noted that the alternate minor conformation of Fmoc makes interactions with M721 and G722. These additional hydrophobic interactions are likely to be at least partly responsible for the enhanced affinity of P6 *vs.* P7 for ScRR1.

The phenylglycine (Phg) residue at position 2 was shown to form intramolecular contacts with the major conformation of Fmoc and L^3^. Also, the L^3^ residue was shown to contact the major conformation of Fmoc, G722, T725, and S726 of the protein, and partially binds in the subsite 1. The carboxyl group of D^4^ was shown to make a weak hydrogen bond with the Cha^5^ amide nitrogen, to interact with the D^6^ side chain, and to make an ion pair interaction with K723. The Cha side chain at position 5 was observed to make contact with K693 only. In P7 and P6, the Cα atoms of D^6^ are 2.6 Å apart. The carboxyl group of D^6^ in P6 was shown to point towards the protein and to make an intramolecular hydrogen bond with the side chain of D^4^ that P7 lacks due to altered side-chain conformations. However, the aromatic F^7^ residue and the terminal carboxylate were shown to bind almost identically within subsite 2 in both structures (Figure 9).

#### 4.2.4. Cyclic Peptides

**Figure 10:**
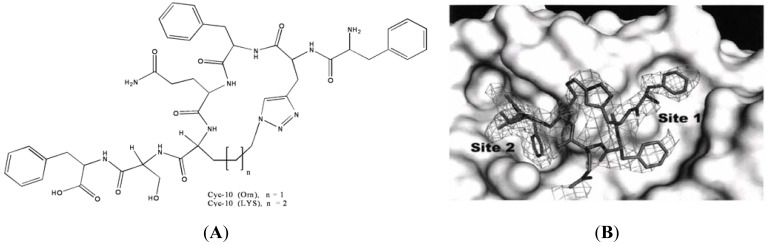
**Cyc 10** (**A**) Cyc 10 structure (**B**) Mode of binding of Cyc 10 to ScRR1.

In another study, we rationally designed a cyclic peptide that was synthesized using click chemistry with the use of a triazole group by the Cooperman group. Based on the structure of ScRR1 complexed with ScR2-pep, ScR4-pep, P7 and P6, the binding site was created. In this study the best fit molecules were docked and energy minimized with the SURFLUX module in SYBYL8.0. This led to the design of [1,2,3]-triazolyl containing cyclic peptides (Figure 10A) with the potential of very tight binding to him RR1. The Cyc 10 shows a P7-like IC50 value in inhibiting hRR.

In this study, a 2.7 Å resolution crystal structure of Cyc10 bound to ScRR1 (Figure 10B) [99] shows two strong interaction sites, denoted subsites 1 and 2, separated by a spacer region which interacts weakly with the inhibitor. Subsite 1 is predominantly hydrophobic and interacts with the N-terminus up until residue three. We showed that the hydrophobic pocket is well occupied by two phenylalanines. Also, subsite 2 is partially polar/hydrophobic and has strong interactions with the Phe and Ser. We observed that the cyclization via the triazole ring connects the two sites; thus rigidifying the turn between residues 2–5 [^1^PhePraPheGlnLysSerPhe^7^] and enhancing the interactions in the spacer region. We showed from the structure of Cyc10 that some side-chains do not interact extensively with R1. Removing these groups may have little impact on binding, while reducing the molecular weight. Also, efforts will be made to enhance the interactions at subsite 1 by the introduction of hydrophobic groups at the N-terminal and altering the spacer region.

It is an interesting question as to if large drug molecules such as the peptide-based molecules described above that defy Lipinski's limits will be able to have *in vivo* efficacies. Although the *in vivo* efficacies of the peptidomimetic/peptide-based inhibitors have not been reported in the literature, they have been shown to have reasonable LD_50_s (Cooperman, personal communications). However, it should be noted that the best efficacies were obtained when the peptide-based inhibitors were conjugated by poly arginine containing peptides. These preliminary results are quite promising and encouraging for the development of a new class of anticancer agents.

Experiments conducted by the Cooperman group and our recent joint efforts show that the P-site is a good drug target for anti-cancer therapy. Molecules targeting the P-site will interfere with the quaternary structure of RR via specific protein-protein interactions, which enable the development of highly specific inhibitors that do not suffer the lack of specificity associated with nucleoside-based analogs that target the A-site, S-site and C-site of RR. Moreover, the P-site consists of two well formed subsites called site 1 and site 2. These two sites will be good candidates for the fragment-based drug design approach.

### 4.3. Inhibitors of the Small Subunit of RR

Although the focus of this review is the inhibition of RR1, we would like to briefly describe some of the efforts involving RR2 inhibition. The inhibitors of the RR2 subunit are either radical scavengers or iron chelators. Hydroxyurea (HU) is the best characterized tyrosyl radical scavenger and has been used to treat various neoplastic and non-neoplastic conditions [100,101]. Its action is reversible and is restricted to S-phase of the cell cycle. Hydroxyurea is used to treat a wide variety of neoplasms, including primary brain cancer, renal cell carcinoma, melanoma, breast cancer, chronic myeloproliferative disorders and chronic myeloid leukemia [102,103]. Recently its therapeutic spectrum has been expanded to treat non-neoplastic diseases such as sickle cell anemia. Hydroxyurea has limited clinical effectiveness as an anticancer drug because of its relatively short half-life and its low affinity towards RR2 in humans.

Another class of inhibitors currently under consideration is the iron chelators [104]. Since tyrosyl radical generation in the RR2 subunit requires iron, iron chelators are considered as one of the most potent inhibitors of RR activity. Deferoxamine is an iron chelator that has shown promise in cancer therapy [105]. It has been shown to inhibit RR activity by reducing the intracellular pool of iron, rather than directly attacking the tyrosyl radical of RR2 [106,107]. Thiosemicarbazones are another group of iron chelators that inactivate RR2. Of these, triapine (3-aminopyridine-2-carboxaldehyde thiosemicarbazone, 3-AP) is the most promising iron chealtor due to its low toxicity. 3-AP has shown higher inhibitory potency over HU and F_2_dCDP in a wide variety of cancer cell lines [108]. Futhermore, cells exposed with 3-AP appear to have enhanced chemoradiosensitivity after treatment with radiation and/or cisplatinum [109]. Currently, 3-AP is undergoing extensive clinical trials to treat cancers as either a monotherapy or a combination therapy [110-114].

## 5. RR the Biomarker

Recent pre-clincial and clinical studies suggest that the individual subunits of RR can be considered as potential biomarkers for predicting outcomes in cancer patients [115]. RR1 subunit overexpression is involved in the suppression of tumour development and metastasis through a variety of mechanisms [116-118]. Tumor suppressor effect of RR1 is associated with increased expression of PTEN, a phosphatase. PTEN dephosphorylate focal adhesion kinase, preventing tumor metastasis [119]. In several pre-clinical studies, increased RR1 expression levels have been observed in breast adenocarcinoma and lung cancer cell lines that are refractory to F_2_CDP [120,121]. Several clinical studies have also shown the promise of the predictive value of RR1 expression levels [122]. Cancer patients who were treated with F_2_CDP having low levels of RR1 had better survival rates than those patients who had undergone surgery alone. In contrast, the overexpression of the R2 subunit correlates to increased tumourogenic potential, enhanced invasiveness and celleular transformation [123-125]. Interestingly, the overexpression of the p53R2 subunit has similar effects to R1 [126]. Therefore, therapeutic decision making based on RR levels, especially the RR1 expression hold promise not only in identifying correct combinations of anti-cancer agents for chemotherapy but also for improving the clinical outcome of cancer patients.

## 6. Conclusions

Ribonucleotide reductase is a well-established cancer and antiviral target. It is emerging as a master enzyme due to its role in maintaining balanced dNTP pools. In a recent review, it has been noted that hRR is a useful biomarker in cancer therapy similar to BRCA1 [115], and recent data has shown that hRR1 expression can be used as a useful biomarker for the selection of agents active in the treatment of cancer. A number of clinical trials have shown that patients with tumors expressing low levels of hRR1 have increased progression-free and overall survival when treated with regimens that included gemcitabine [115]. So far, hRR1 has four known druggable sites that include: the A-site, S-site, C-site and the P-site. The clinically used nucleoside analogues such as clofarabine target the A-, S- and C-sites while F_2_dCDP targets the C-site. As human RR1 is thought to communicate with several protein molecules in the cell, new druggable sites are likely to emerge. In spite of the fact that human RR1 is a rich drug target, only a limited effort has been devoted to developing new drugs against it. As we begin to understand more about how this complicated molecule functions, a greater effort should be devoted to develop new classes of chemotherapies that target hRR1.
